# Longitudinal association of dietary habits and the risk of cardiovascular disease among Iranian population between 2001 and 2013: the Isfahan Cohort Study

**DOI:** 10.1038/s41598-023-32387-w

**Published:** 2023-04-01

**Authors:** Maryam Mohseni, Noushin Mohammadifard, Razieh Hassannejad, Mahnaz Aghabozorgi, Fatemeh Shirani, Masoumeh Sadeghi, Hamidreza Roohafza, Nizal Sarrafzadegan

**Affiliations:** 1 Department of Community Nutrition, School of Nutrition and Food Science, Isfahan University of Medical Sciences, Isfahan, Iran; 2 Isfahan Cardiovascular Research Center, Cardiovascular Research Institute, Isfahan University of Medical Sciences, Isfahan, Iran; 3 Interventional Cardiology Research Center, Cardiovascular Research Institute, Isfahan University of Medical Sciences, Isfahan, Iran; 4 Senior Endocrine Dietitian and Credentialed Diabetes Educator, Departments of Dietetics, and Endocrinology and Diabetes, Fiona Stanley and Fremantle Hospitals Group, Perth, Australia; 5 Food Security Research Center, Isfahan University of Medical Sciences, Isfahan, Iran; 6 Cardiac Rehabilitation Research Center, Cardiovascular Research Institute, Isfahan University of Medical Sciences, Isfahan, Iran; 7 Heart Failure Research Center, Cardiovascular Research Institute, Isfahan University of Medical Sciences, Isfahan, Iran; 8 School of Population and Public Health, Faculty of Medicine, University of British Columbia, Vancouver, Canada

**Keywords:** Cardiovascular diseases, Nutrition

## Abstract

There has been a steady rise in the incidence of cardiovascular disease (CVD) in the Iranian population. The aim of this study is to investigate the association between Global Dietary Index (GDI) and CVD risk among the Iranian adult population. This study was conducted based on Isfahan Cohort Study, a longitudinal study that collected data between 2001 and 2013 on 6405 adults. Dietary intakes were assessed by a validated food frequency questionnaire to calculate GDI. All participants were followed every two years by phone call to ask about death, any hospitalization, or cardiovascular events to examine CVD events. The Average age of participants was 50.70 ± 11.63 and the median of GDI score was 1 (IQR: 0.29). A total of 751 CVD events (1.4 incidence rate, per 100 person-year) occurred during 52,704 person-years of follow-up. One-unit GDI increase was associated with a higher risk of MI by 72% (HR: 1.72; 95% CI 1.04–2.84), stroke by 76% (HR: 1.76; 95% CI 1.09–2.85) and CVD by 30% (HR: 1.48; 95% CI 1.02–2.65). In addition, a one-unit GDI increase was associated with a higher risk of coronary heart disease more than 2 times (HR: 2.32; 95% CI 1.50–3.60) and CVD mortality and all-cause mortality over than 3 times [(HR: 3.65; 95% CI 1.90–7.01) and (HR: 3.10; 95% CI 1.90–5.06), respectively]. Higher GDI had a significant relationship with the increased risk of CVD events and all-cause mortality. Further epidemiological studies in other populations are suggested to confirm our findings.

## Introduction

In recent decades, cardiovascular diseases (CVD) are reported to be one the leading causes of mortality worldwide^1,2^. It has been estimated that 80% of the global burden of CVD occur in low-income and middle-income countries and its incidence has been increasing in these countries^3^. Iran was among countries with the highest age-standardized prevalence of CVD in 2015^4^. This may be due to the unique dietary pattern of the Iranian population^5^. The Iranian diet is highly loaded with hydrogenated fats and refined grains^6^. Current evidence suggest that dietary factors have a major role in prevention and treatment of CVD^7^.

The association of various nutrients and foods with CVD events extensively investigated in several studies around the world^8–10^. However, due to the interaction of nutrients and foods, overall diet might be more informative than using only single nutrient or food^11^. Overall diet can be examined by dietary patterns such as Mediterranean and Western dietary patterns and various dietary scores including the Diet Quality Index (DQI), the Dietary Diversity Score (DDS), Healthy Eating Index (HEI)^12^ and the global dietary index (GDI)^13^. Several studies have reported the relationship between dietary scores and CVD risk factors^14,15^. In addition, the link between diverse nutrients and foods with the risk of CVD has been established in Iran^16–21^. However, one study has indicated the association of Mediterranean dietary patterns and CVD mortality, but not other CVD events^22^.

We used GDI to examine diet quality since it is one of dietary scores^13^ attributed based on Cholesterol-Saturated Fat Index, an index of atherogenicity of food^23^, for specific food as well as recommendations from the American Heart Association and the National Cholesterol Education Program^24^. It was ranged from zero to two which represents atherogenicity of diet through 29 items and categorized in seven food groups including fast food; vegetables and fruit; legumes, chicken, soy protein and fish; sweets; butter, hydrogenated oil, animal fats and ghee; egg, whole dairy products and meat; olive and non-hydrogenated oil in Iran. All food groups within the GDI have atherogenic or antiatherogenic effects. The atherogenic food groups got higher scores and antiatherogenic got a smaller score. Hence, the lower GDI indicates healthier dietary behaviour^25^. The GDI developed based on a simplified food frequency questionnaire (FFQ)^26^. The food items in the questionnaire were mostly consistent with the components of this score.

Due to high incidence of CVD along with the particular dietary behaviors of Iranians, as there is no study evaluating the relationship between diet quality and the risk of CVD and all-cause mortality this study, in the framework of longitudinal study of Isfahan cohort study (ICS), aimed to investigate the association of GDI as an index showing the dietary habits and the risk of CVD and all-cause mortality among Iranian adults population.

## Subjects and methods

### Study population

This longitudinal study was conducted based on ICS that is a population-based longitudinal study on 6405 adults, living in urban and rural areas of three districts (2153 from Isfahan, 1028 from Najaf-Abad, and 3323 from Arak) in central Iran. All the methods were performed in accordance with the declaration of Helsinki. Informed consent was obtained from all subjects before starting the study.

The subjects of ICS was participants of Isfahan Healthy Heart Program (IHHP) baseline survey^27,28^. IHHP was a community trial for prevention and control of CVD. Our inclusion criteria was adults aged 35 years and over, having no chronic diseases such as cancers, kidney failure, liver failure and gastrointestinal disorders, non-pregnant and non-lactating status, no consumption of food supplements. Recruitment was conducted from January to September 2001. Multistage random sampling was used for selecting participants to reflect age, sex and urban/rural distribution of the community. Laboratory measurements, physical examinations, and interviews were conducted at baseline and repeated in 2007 and 2013.

We excluded from the analysis those with a previous history of CVD (n = 181) and also those who dropped out before the first follow-up (n = 892). Finally, among initial participants, 5432 participants were recruited in the study. All participants were followed every two years by phone call to ask about death, any hospitalization or cardiovascular events to examine CVD events including myocardial infarction (MI), stroke, coronary heart disease (CHD), major CVD events, CVD death and also all-cause mortality. Subjects were censored if reported CVD events in repeated measurements or any biannually follow up interviews. Study design and details of subjects’ recruitment and data collection methods have been reported previously^29^.

### Data collection

Interviews were carried out by trained health professionals at baseline and two repeated measurements for obtaining the participants’ general characteristics including socioeconomic and demographic status, life style behaviours (diet, smoking and physical activity) using a validated questionnaires^26,30,31^. Smoking status was defined in three category including smoker for those who smoked at least one cigarette per day during the study; ex-smoker for those who smoked previously at least one cigarette per day, and non-smoker. Physical activity was assessed by international physical activity questionnaire (IPAQ) which was validated in Iranian population^31^. The data on physical activity was expressed as metabolic equivalent task minutes per week, were obtained through a questionnaire that included questions on recreational activities, sport and leisure time physical activity. Participants were requested to think about all the vigorous and moderate activities they had executed in the last 7 days, including the number of days per week and the time spent on these activities^31^. Height was measured without shoes to the nearest 0.5 cm, and weight was measured without shoes and with light clothing with a precision of 0.1 kg using a Seca scale. The WC was measured at the horizontal plane midway between the lowest rib and the upper border of the iliac crest at the end of normal inspiration/expiration. Body mass index (BMI) was calculated as weight in kilograms divided by square of height in meters (kg/m^2^). Blood pressure was measured manually by a trained operator using a mercury sphygmomanometer according to standard protocol^32^, twice from right and left arms in sitting position after 5 min of rest. We kept the blood pressure measurement environment silent to hear Korotkoff sound. The first Korotkoff sound was recorded as the systolic blood pressure (SBP), and the disappearance of the sounds (V phase) was considered the diastolic blood pressure (DBP). The mean of the two blood pressure readings on the arm with higher BP was used in the analyses. Having blood pressure ≥ 140/90 mmHg (based on average of two measurements) or the use of antihypertensive medications was defined as hypertension^33^. Blood samples were gathered after 12 h fasting status and stored in laboratory of Isfahan Cardiovascular Research Institute at − 70 °C. Fasting blood glucose (FBG), serum total cholesterol (TC) and triglycerides (TG) were measured by the enzymatic method by a Hitachi auto-analyzer (Hamburg-Eppendorf Hamburg, Germany) using special kits (Immunodiagnostic, Frankfurt, Germany). High-density lipoprotein cholesterol (HDL-C) was determined enzymatically after precipitating other lipoproteins with dextran sulfate magnesium chloride^34^. The Friedewald formula was utilized to calculate LDL-C in individuals with TG < 400 mg/dL. However, direct measurement of LDL-C was performed with a turbidimetric method for those with TG ≥ 400 mg/dL^35^. Those individuals with fasting plasma glucose levels of ≥ 126 mg/dL or on insulin or oral hypoglycemic agents were considered as having diabetes mellitus^36^. Hypercholesterolemia was defined as having serum total cholesterol levels ≥ 200 mg/dL or hypolipidemic agent’s use^37^.

### Dietary assessment

Usual dietary intakes were measured with the use of a validated 48-item, FFQ^26^. Participants reported the frequency of consumption of food items over the preceding year on a daily, weekly or monthly basis. Subjects were also requested to choose the 'never/seldom' response if they never or rarely consumed a given food item. Seldom and never were calculated as 'zero '.

To calculate the mean weekly consumption of all food items, the frequency consumption was multiplied by 7 if the participants reported daily consumption and divided by 4 if they reported monthly consumption in their FFQs. The FFQs were completed by trained interviewers in a face-to-face interview and GDI was calculated. GDI is representing the average of twenty-nine frequency questions in seven categories including fast food (4 items); vegetables and fruit (7 items); legumes, chicken, soy protein or fish (4 items); all kinds of sweets (6 items); butter, hydrogenated oil, animal fats, or ghee (4 items); egg, whole dairy products, or meat (4 items); and olive and non-hydrogenated oil (2 items). Each of question’s category was scored 2, 1 or 0 according to frequency of use of food parameters and based on nutritional values. For the question about healthy food items such as fruit and vegetables, dairy products or beans and fish a less frequency of consumption scored higher and more frequency intake scored lower. For the questions about unhealthy food items such as fast food or sweets a higher frequency of use received a higher score. Then, the scores to individual questions were summed and a total score was calculated. A smaller GDI indicates healthier diet which is rich in fruit and vegetables, beans, fish, non-hydrogenated oil and olive oil. Higher scores on the GDI represent diets with higher intake of fast foods, sweets, red meat and hydrogenated oils, whole dairy products. More detailed description of the method has been published previously^25^. The definition of GDI was presented in Table 1. A validation study comparing FFQ with one 24-h dietary recall and 2 days dietary record revealed good relative reproducibility and validity of FFQ. The Spearman correlation coefficients between examined FFQ and reference method ranged from 0.113 to 0.480 for seven categories included in GDI definition. The intraclass correlation coefficient (ICC) presented the reproducibility of FFQ varied from 0.51 to 0.69^24^. The alpha Cronbach coefficient was 0.79 for the FFQ.

**Table 1:** Global dietary index definition.

	Items	Number of items	Score		
			0	1	2
1	How many times per week do you eat fast foods?	4	0–1	2–3	4 ≤
2	How many serving of fruits or vegetables do you eat in a week?	7	28 ≤	14–27	< 14
3	How many times per week do you eat beans, chicken, soya protein or fish?	4	3 ≤	1–2	< 1
4	How many times per week do you eat sweets?	6	0–1	2–3	4 ≤
5	How many times per week do you eat hydrogenated oil, ghee, animal fats or butter?	4	0–1	2–3	4 ≤
6	How many times per week do you eat meat, egg or whole dairy products?	4	0–1	2–3	4 ≤
7	How many times per week do you eat non-hydrogenated oil, olive oil?	2	7 ≤	5–6	< 5

### Ascertainment of CVD events

We followed participants until the incidence of the first major CVD events or death. The median duration of follow-up was 11.25 (interquartile range: 7.75–12.25) years. Trained nurses carried out interviews biannually by telephone call with the subjects or their families. In this interview, trained nurses asked about the subjects’ death, any hospitalization or cardiovascular events, its reason (with emphasize on coronary and cerebrovascular diseases), the physician diagnosis and the hospital’s name^29^. Related documents about reported events were collected through several approaches including medical records, hospital records, death certificates, and verbal autopsies by trained nurses. The specialists’ outcome adjudication panel consisted of four cardiologists and two neurologists evaluated the documents related to each event^29^.

In the present study, we defined cardiovascular events, as fatal and non-fatal myocardial infarction (MI), fatal and non-fatal stroke (which was defined as death from cerebrovascular disease) and sudden cardiac death (SCD)^38^. The MI was defined based on the presence of at least two of the following criteria including typical chest pain lasting more than 30 min, ST elevation > 0.1 mV in at least 2 adjacent electrocardiograph leads, and an increase in serum level of cardiac biomarkers.

SCD was defined as death within 1 h of onset, a witnessed cardiac arrest, or abrupt collapse not preceded by > 1 h of symptoms. Stroke was defined according to the WHO stroke definition^39^, i.e. stroke was defined as a rapid-onset focal neurological disorder persisting at least 24 h that had probable vascular origin. CHD included fatal and non-fatal MI and SCD.

### Statistical analysis

Descriptive statistics were expressed as mean (SD) and percent for quantitative and qualitative variables, respectively. The comparisons of participants’ frequency across CVD and also quartiles of GDI were assessed by Chi-square test. We applied t-test to compare mean of variables across CVD occurrence. Mann–Whitney non-parametric test was alternative one when the assumption of normality was not met. One-way analysis of variance was used to compare mean of variables across quartiles of GDI. Kruskal–Wallis non-parametric test or Brown-Forsyth robust test were alternative ones when the assumptions of one-way analysis of variance were not met. Jonckheere-Terpstra test was utilized to evaluate median trend of food and food groups intakes across quartiles of GDI.

The hazard ratios and 95% confidence intervals (CI) for cardiovascular events across quartiles of baseline GDI as an independent were estimated based on Cox proportional hazards regression models in total population and also stratified based on sex.

Further analysis was done by applying mixed-Cox regression to determine the longitudinal associations of GDI over time as an independent variable (as quantitative variable) with CVD events and all-cause mortality. This model takes into account the random effect to consider the repeated measurement of covariates as the time-varying covariate to control unmeasured confounders during the long follow-up period (13 years). Age (continuous), sex (men/women), education (illiterate, primary school and higher than primary school), residence area (urban/rural), smoking status (never, ex-smoker, current smoker), daily physical activity (continuous, METs-min/day), aspirin use (yes/no), menopausal status in women (yes/no), body max index (BMI), hypertensin (yes/no) and diabetes mellitus (yes/no) were adjusted in modeling process through three hierarchical steps. We further applied stratified analysis based on sex using mixed-Cox regression to examine GDI association with various events.

STATA software, version 14 and RStudio software were used to perform statistical analyses. P-value (P) < 0.05 (two-tailed) was considered as statistically significant.

### Ethical approval

The study protocol was approved by the Isfahan Cardiovascular Research Institute ethics committee, a World Health Organization (WHO) collaborating center.

## Results

The median of follow-up was 11.25 (interquartile range: 7.75–12.25) years. The study recognized 156 cases for MI and 245 for CHD per 55,017 person-years of follow-up, 157cases for stroke per 55,048 person-years of follow-up, 751 new cases for CVD per 52,704 person-years of follow-up, 179 CVD mortality and 458 all-cause per 55,618 person-years of follow-up.

Baseline characteristics in participants by quartiles of GDI are provided in Table 5 in Appendix. Participants in lower quartiles of GDI, were significantly older, less likely to be men, less illiterate, less current smoker, and also had lower physical activity, higher level of SBP, DBP, TC, TG and LDL-C and were more suffering from diabetes mellitus and hypertension P (all P-values < 0.001) (Table 5 in Appendix).

General characteristics of participants at study baseline according to CVD events are presented in Table 2. Individuals with CVD were significantly older (P < 0.001), had lower physical activity (P < 0.001), were less likely to be women (P = 0.002) and being educated (P < 0.001). In addition, those with CVD had significant higher mean of BMI, SBP, DBP, total cholesterol, triglyceride and LDL-C (all P-values < 0.001). They also were likely to be more hypertensive and diabetic (all less than 0.001).

**Table 2:** Baseline characteristics in participants by cardiovascular disease.

	CVD (+) N = 751	CVD (−) N = 4681	P value
Mean GDI	0.97 (0.28)	0.98 (0.27)	< 0.341^1^
Age (years)	57.37 (11.67)	49.63 (11.26)	< 0.001^1^
Physical activity (METs-min/day)	12.93 (9.37)	14.80 (9.08)	< 0.001^2^
Men (%)	53.9	47.9	0.002^3^
Illiterate (%)	47	34.7	< 0.001^3^
Family history of CVD (%)	6.8	5.1	0.06^3^
Current smoker (%)	18.3	15.9	0.098^3^
BMI (kg/m^2^)	27.31 (4.67)	26.60 (4.41)	< 0.001^1^
SBP (mmHg)	132.82 (24.01)	119.83 (19.81)	< 0.001^1^
DBP (mmHg)	82.98 (12.74)	77.65 (11.17)	< 0.001^1^
Total cholesterol (mg/dL)	228.20 (55.58)	211.86 (51.35)	< 0.001^1^
Triglycerides (mg/dL)	216.55 (114.89)	187.23 (100.70)	< 0.001^2^
LDL-C (mg/dL)	137.99 (45.73)	127.50 (42.90)	< 0.001^1^
HDL-C (mg/dL)	46.90 (10.55)	46.92 (10.34)	0.97^1^
Diabetes mellitus (%)	24.4	8.9	< 0.001^3^
Hypertension (%)	50.6	25.7	< 0.001^3^

Data are expressed as mean (SD) or percent.

*GDI* global dietary index, *BMI* body mass index, *SBP* systolic blood pressure, *DBP* diastolic blood pressure, *LDL-C* low density lipoprotein cholesterol, *HDL-C* high density lipoprotein cholesterol.

^1^Obtained from t-test.

^2^Obtained from Mann-Withney test.

^3^Frequency comparison obtained from Chi-Square.

Dietary intakes in participants by quartiles of GDI are shown in Table 6 in Appendix. Higher quartiles of GDI had higher intake of hydrogenated vegetable oils (HVOs), read meat, cereals, sweets and soft drinks, fast food and dairy products and had lower intake of non- HVOs, fruits and vegetables (all P-values < 0.001).

Compared with participants without CVD events, individuals with occurred CVD events consumed higher amounts of HVOs, red meat, high fat dairy products, fast foods, sweets and soft drinks (P < 0.001 for all) and lower amounts of non-HVOs (P = 0.014) (Table 3).

**Table 3:** Dietary intakes in participants with cardiovascular disease.

Dietary intakes (times/week)	CVD (+)	CVD (−)	P-value
Hydrogenated vegetable oil	7 (7–14)	7 (4–14)	< 0.001
Non-hydrogenated vegetable oil	0.25 (0–4)	0.50 (0–7)	0.014
Red meat	4 (2–7)	3 (2–7)	< 0.001
Fish	0.2 (0–0.5)	0.2 (0–0.5)	0.672
High fat dairy products	0 (0–0.50)	0 (0–0)	< 0.001
Fruits and vegetables	14 (8–14)	14 (8–14)	0.517
Fast foods	0 (0–0.7)	0 (0–0.5)	< 0.001
Cereals	24 (21–28)	24 (21–28)	0.452
Sweets & soft drinks	2.0 (0.7–4.2)	1.7 (0.5–3.7)	< 0.001

Data are expressed as median (Q1–Q3) due to non-normality.

Comparison was obtained from Mann–Whitney test.

Multiple adjusted relative risk of CVD by GDI quartiles are presented in Table 7 in Appendix. After adjusting for all confounders a significant higher risk of CHD was found amongst the individuals in higher quartiles of GDI [HR (95% CI) 1.47 (0.93–2.31); P = 0.03]. There was no significant association between GDI and other events.

Multivariable adjusted HR for CVD according to GDI are presented in Table 4. After full adjustment, one unit GDI increase was associated with the higher risk of MI, stroke, CVD, by 72% [HR: 1.72; 95% CI (1.04–2.84); P = 0.033], 76% [HR: 1.76; 95% CI (1.09–2.85); P = 0.022] and 30% [HR: 1.30; 95% CI (1.02–1.65); P = 0.033], respectively and higher risk of CHD more than 2 times [HR: 2.32; 95% CI (1.50–3.60); P < 0.001] and CVD mortality and all-cause mortality over than 3 times [HR: 3.65; 95% CI (1.90–7.01); P < 0.001; and (HR: 3.10; 95% CI 1.90–5.06); P < 0.001, respectively] in total population.

**Table 4:** Multiple adjusted hazard ratio and 95% confidence interval of cardiovascular diseases by global dietary index.

GDI	Total^a^		Men^b^		Women^b^	
	HR (95% CI)	P	HR (95% CI)	P	HR (95% CI)	P
MI						
Crude model	1.25 (0.81–1.94)	0.320	1.14 (0.68–1.92)	0.620	1.47 (0.69–3.11)	0.320
Model 1	1.33 (0.83–2.12)	0.230	1.18 (0.70–2.00)	0.540	1.41 (0.61–3.25)	0.420
Model 2	1.72 (1.04–2.84)	0.033	1.49 (0.80–2.77)	0.210	2.18 (0.93–5.10)	0.074
CHD						
Crude model	1.66 (1.15–2.42)	0.007	1.47 (0.93–2.31)	0.096	2.05 (1.04–4.02)	0.037
Model 1	1.75 (1.20–2.54)	0.004	1.56 (0.99–2.47)	0.055	1.84 (0.85–3.97)	0.120
Model 2	2.32 (1.50–3.60)	< 0.001	1.93 (1.13–3.30)	0.016	3.20 (1.60–6.40)	0.001
Stroke						
Crude model	1.32 (0.85–2.06)	0.210	1.42 (0.74–2.70)	0.290	1.24 (0.67–2.30)	0.490
Model 1	1.40 (0.90–2.18)	0.140	1.39 (0.68–2.84)	0.370	1.31 (0.70–2.44)	0.400
Model 2	1.76 (1.09–2.85)	0.022	1.90 (0.93–3.84)	0.076	1.60 (0.67–3.80)	0.290
CVD						
Crude model	0.96 (0.79–1.16)	0.67	0.93 (0.71–1.20)	0.570	0.99 (0.75–1.32)	0.970
Model 1	1.02 (0.83–1.25)	0.840	0.95 (0.73–1.24)	0.720	1.06 (0.78–1.44)	0.720
Model 2	1.30 (1.02–1.65)	0.033	1.23 (0.88–1.71)	0.220	1.43 (1.00–2.05)	0.052
CVD mortality						
Crude model	2.58 (1.46–4.58)	0.001	2.74 (1.34–5.63)	0.006	2.35 (0.97–5.73)	0.059
Model 1	3.23 (1.83–5.71)	< 0.001	3.58 (1.65–7.75)	0.001	2.51 (0.90–7.04)	0.079
Model 2	3.65 (1.90–7.01)	< 0.001	3.74 (1.58–8.86)	0.003	3.07 (1.16–8.14)	0.024
All-cause mortality						
Crude model	2.72 (1.76–4.19)	< 0.001	2.45 (1.28–4.68)	0.007	2.49 (1.25–4.94)	0.009
Model 1	3.32 (2.16–5.11)	< 0001	3.99 (2.46–6.47)	< 0.001	2.97 (1.73–5.11)	< 0.001
Model 2	3.10 (1.90–5.06)	< 0.001	3.45 (2.00–5.97)	< 0.001	3.16 (1.54–6.42)	0.001

*GDI* global dietary index, *HR (95% CI)* hazard ratio (95% confidence interval), *MI* myocardial infarction, *CHD* coronary heart disease, *CVD* cardiovascular disease.

^a^Model 1: Adjusted for age (year) and sex (men/women); model 2: Additionally adjusted for education (illiterate/primary school/more than primary school), residency (urban/rural), smoking status (never/past/current smoker), daily physical activity (METs-min/day), aspirin use (yes/no), body mass index (kg/m^2^), hypertension (yes/no) and diabetes mellitus (yes/no) and also post menopause in women (yes/no) only in women.

^b^Model 1: Adjusted for age (yr); model 2: Additionally adjusted for education (illiterate/primary school/more than primary school), residency (urban/rural), smoking status (never/past/current smoker), daily physical activity (METs-min/day), aspirin use (yes/no), body mass index (kg/m^2^), hypertension (yes/no) and diabetes mellitus (yes/no) and also post menopause in women (yes/no) only in women.

We did not find any significant relationship between CVD risk and GDI quartiles in men and women (Table 8 and Table 9 in Appendix, respectively). Sex base analysis showed that one unit GDI increasing was associated with higher CHD risk about 2 times in men and 3 times in women [(HR: 1.93; 95% CI 1.13–3.30) and (HR: 3.20; 95% CI (1.60–6.40), respectively] and more than 3 times risk of CVD mortality [(HR: 3.74; 95% CI 1.58–8.86) in men and (HR: 3.07; 95% CI 1.16–8.14) in women] and all-cause mortality [(HR: 3.45; 95% CI 2.00–5.97) in men and (HR: 3.15; 95% CI 1.54–6.42) in women]. However, there was no significant relationship between MI, stroke and CVD risk in both sexes.

## Discussion

Our findings through this cohort study showed that increasing the GDI was significantly associated with the higher risk of all CVD events including MI, CHD, stroke, CVD and CVD mortality as well as all-cause mortality in total population, increasing the GDI had significant relationship with CHD, CVD mortality and all-cause mortality in both men and women, but not with increased risk of MI, stroke and CVD.

The results represent that the mean BMI, systolic and diastolic blood pressure, and also hypertension and diabetes mellitus were higher in individuals who have been occurred CVD than those without occurrence of CVD. This finding may be due to the fact that those with underlying diseases and risk factors are more likely to adhere to healthier diet^40^.

In this study we found an inverse association between GDI score characterized with high amount of animal product and low amount of fruit and vegetables and CHD which is consistent with the result of a meta-analysis in which 409 780 participants in twelve prospective studies was involved for examining the association between principal component analysis-defined dietary patterns and CHD risk that shows an inverse association between CHD risk and prudent/healthy dietary pattern^41^. Also several studies have shown that the lower CHD rate in Japan and Mediterranean region might be due to their dietary pattern similar to prudent diet with low amount of animal product and high amount of fruit and vegetables and whole grains^42,43^. Greater adherence to the prudent pattern contributed to smoothing weight maintenance, endothelial dysfunction and markers of inflammation which can be the cause of that results^44^. Theincreasing GDI was inversely associated with CVD mortality as well as all-cause-mortality in total population which is consistent with some previous studies^22,45–48^. Heidemann et al. in a prospective cohort found that greater adherence to the prudent pattern could diminish the risk of CVD and all-cause mortality which is in line with our findings, whereas among initially healthy women, greater adherence to the Western pattern might increase the risk of them^49^. Additionally a meta-analysis including 13 prospective studies discussed an inverse association between a prudent/healthy dietary pattern and CVD mortality, however it indicates that Western/unhealthy dietary pattern was not relared to all-cause and stroke mortality^50^. The inverse association between a mediterranean diet as a healthy diet and the risk of CVD mortality was indicated in previous study on the ICS data^22^. On the other hand, Western dietary pattern in Osler’s study and animal fat diet in Shimazu’s study which show high GDI score had no relationship with all-cause mortality and CVD mortality respectively^45^. Our results showed that the occurrence of CVD events was associated with adherence to healthy diet and lower scores of GDI. This finding is aligned with a systematic review and meta-analysis of twenty-two observational studies which supported the evidence of protective effect of prudent dietary pattern for CVD, while it did not show a direct association between adherence to unhealthy dietary patterns and CVD incidence^51^. Our findings showed that lower GDI score is associated with lower risk of MI and stroke. Consistently, 10 years follow up in a cohort study of 32,921 women showed that Better adherence to a Mediterranean diet was associated with lower risk of MI, HF and ischemic stroke^52^.

The result of this study could be explained by various biological mechanisms. A prudent/healthy dietary pattern showing a lower GDI score diet with high intakes of cereals, fish and poultry, olive oils and also fresh fruits and vegetables. The consumption of red meat, fast food, sweets and soft drink and HVOs which are the components of western and animal fat diets were indicating higher GDI^45,47^. The more intake of fruit, vegetables, whole grains and legumes, the more consumption of fiber we have, which can cause protective effect against CVD. Furthermore, several systematic reviews have shown that the consumption of vegetables (> 2 servings/day, 200 g) and fruits (> 2 servings/day, 200 g) significantly reduces the risk of CHD and stroke^53,54^. Also fruits and vegetables have plenteous antioxidants such as flavonoids, vitamin C and K and folates that might affect the decrease in CVD risk^51^. Also the origins of PUFA (n-3 fatty acid) such as oily fish and nuts reduce the risk of CHD^55^.The sex-based analysis showed that increasing the GDI had significant relationship with CHD, CVD mortality and all-cause mortality in both men and women, but not with increased risk of MI, stroke and CVD. Similar to our result a prospective cohort study among 23 929 adult men and women showed an inverse association between Mediterranean diet score and CHD incidence and mortality^56^. A meta-analysis study showed an inverse association of a prudent/healthy dietary pattern which was characterized by high intake of vegetables, fruit, soy products, potatoes, seaweed, mushrooms, and fish with all-cause mortality in 36,737 men and 44,983 women^57^. A prospective cohort study during 5,291,518 person-years of follow-up indicates dietary patterns with a higher proinflammatory potential (unhealthy diet) were associated with higher CVD risk among men and women^58^ which is conflicting to our results. In explaining this result, we can refer to the GDI which we use for indicating the quality of diet that may be inefficient in sex based stratified analysis with sex stratification in future CVD studied with variable indices could help reach a certain conclusion. We accomplished sensitivity analysis by sex as gender differences have a main role in the occurrence of many diseases, especially CVD. Studies have shown that the risk of MI is higher amongst men and due to the cardio-protective role of estrogen the risk of CVD is higher amongst women after menopause. Also this difference can be due to oxidative stress which is associated to gender and females appear to be less susceptible to oxidative stress^59^.

### Strengths and limitation

Large sample size in this study was a major strength that enhanced the power of analysis. Socioeconomic status of the samples was heterogeneous so the study has encompassed a wide range of dietary intakes. The prospective design in which dietary behaviors were assessed before the incidence of the events, strengthens the causal deduction. Repeated measurement of dietary intake was another strength of the current study. Some limitations of this study need to be verified. Although a validated FFQ was used for dietary data collection, we cannot exclude the existence of misclassification, as is the case in all epidemiologic studies. Moreover, FFQ did not provide data on the portion sizes and energy intake. Therefore, we did not have any data about total energy intake in this study. Reverse causality was another limitation of the current study as other observational cohort studies. Finally, although we adjusted the effect of all potential confounders, some residual confounders might not have affected the findings.

## Conclusion

We concluded that higher GDI score characterized by higher intake of red meat, sweets, soft drinks and fast foods and lower amounts of fruit and vegetables, dairy products, cereal, fish and non-HVO was associated with higher MI, CHD, stroke, CVD, CVD mortality and all-cause death risk in total population as well as the risk of CHD, CVD mortality and all-cause death in both men and women. We did not find any relationship between GDI and risk of MI, stroke and CVD in both sexes. Further well-designed epidemiological studies in other populations are suggested to confirm our findings.

## Data Availability

The datasets used during the current study are available from the corresponding author on reasonable request.

## Appendix

See Tables 5, 6, 7, 8 and 9.

**Table 5:** Baseline characteristics in participants by quartiles of global dietary index.

	Global dietary index				P-value
	Q1 N = 1099	Q2 N = 912	Q3 N = 2558	Q4 N = 863	
Mean GDI	0.56 (0.18)	0.86 (0.08)	1.06 (0.07)	1.34 (0.09)	
Age (years)	53.81 (12.10)	50.80 (11.43)	50.33 (11.54)	47.75 (10.51)	< 0.001^1^
Physical activity (METs-min/day)	13.23 (8.64)	14.21 (9.23)	14.55 (8.91)	16.51 (10.01)	< 0.001^1^
Men (%)	46	48.3	48.4	53.7	0.008^4^
Illiterate (%)	31.3	36.3	40.9	29.9	< 0.001^4^
Family history of CVD (%)	6.9	5.8	4.3	5.9	0.014^4^
Current smoker (%)	20.1	20.6	21.4	27.6	< 0.001^4^
BMI (kg/m^2^)	27.78 (4.27)	26.86 (4.36)	26.38 (4.52)	26.09 (4.36)	< 0.001^2^
SBP (mmHg)	126.49 (21.79)	122.32 (21.34)	120.78 (20.84)	117.19 (18.23)	< 0.001^3^
DBP (mmHg)	80.20 (11.57)	78.68 (11.96)	78.06 (11.62)	76.75 (10.53)	< 0.001^2^
Total cholesterol (mg/dL)	223.36 (52.82)	213.33 (53.53	212.98 (51.83)	206.58 (49.82)	< 0.001^2^
Triglycerides (mg/dL)	210.32 (109.50)	198.59 (115.06)	184.73 (96.13)	178.78 (98.49)	< 0.001^1^
LDL-C (mg/dL)	133.77 (43.51)	126.68 (43.87)	129.31 (43.42)	124.14 (42.37)	< 0.001^2^
HDL-C (mg/dL)	47.53 (10.50)	46.93 (10.42)	46.73 (10.30)	46.69 (10.32)	0.172^2^
Diabetes mellitus (%)	15.9	12.5	9.9	6.4	< 0.001^4^
Hypertension (%)	40	28.6	27.6	20.6	< 0.001^4^

*GDI* global dietary index, *BMI* body mass index, *SBP* systolic blood pressure, *DBP* diastolic blood pressure, *LDL-C* low density lipoprotein cholesterol, *HDL-C* high density lipoprotein cholesterol.

^1^Obtained from Kruskal–Wallis test.

^2^Mean comparison obtained from ANOVA test.

^3^Obtained from Brown-Forsyth test.

^4^Frequency comparison obtained from Chi-Square.

**Table 6:** Dietary intakes in participants by quartiles of global dietary index.

Dietary intakes (times/week)	Global dietary index				
	Q1	Q2	Q3	Q4	P-value^1^
Hydrogenated vegetable oil	2 (0–7)	7 (7–14)	7 (7–14)	7 (7–14)	< 0.001
Non-hydrogenated vegetable oil	7(4.23–7.70)	1 (0–7)	0 (0–1)	0 (0–1)	< 0.001
Red meat	2 (1–3)	3 (2–4)	4 (3–7)	7 (3–7)	< 0.001
Fish	0.23 (0–1)	0.23 (0–0.47)	0 (0–0.23)	0.23 (0–0.47)	< 0.001
Dairy products	0 (0–0)	0 (0–0)	0 (0–1)	0 (0–1)	< 0.001
Fruits and vegetables	14 (14–16)	14 (14–15)	14 (7–14)	10 (7–14)	< 0.001
Fast foods	0 (0–0.47)	0 (0–0.47)	0 (0–0.47)	0.70 (0–2)	< 0.001
Cereals	23 (18–28)	24 (21–28)	25 (21.23–28)	25 (21–28)	< 0.001
Sweets & soft drinks	1.23 (0.47–2.70)	1.47 (0.47–3.23)	1.70 (0.47–3.70)	5.63 (3.23–9)	< 0.001

^1^Data are expressed as median (Q1–Q3) due to non-normality.

Comparison was obtained from Jonckheere-Terpstra test.

**Table 7:** Multiple adjusted relative risk of cardiovascular diseases by global dietary index quartiles.

	Mortality rate		Crude model	Model 1^a^ HR (95% CI)	Model 2^b^ HR (95% CI)
	N	Person-year	HR (95% CI)		
MI					
Q1	32	11,378.8	1	1	1
Q2	33	9263.4	1.26 (0.78–2.06)	1.45 (0.89–2.37)	1.58 (0.96–2.60)
Q3	69	25,566.7	0.96 (0.63–1.45)	1.15 (0.75–1.76)	1.31 (0.84–2.04)
Q4	22	8808.2	0.88 (0.51–1.52)	1.15 (0.67–2)	1.30 (0.74–2.29)
P for trend			0.44	0.77	0.41
CHD					
Q1	48	11,378.8	1	1	1
Q2	42	9263.4	1.08 (0.71–1.63)	1.30 (0.86–1.98)	1.38 (0.91–2.10)
Q3	121	25,566.7	1.12 (0.80–1.57)	1.43 (1.02–2.01)	1.58 (1.11–2.25)
Q4	34	8808.2	0.91 (0.59–1.42)	1.32 (0.85–2.06)	1.47 (0.93–2.31)
P for trend			0.96	0.08	0.03
Stroke					
Q1	38	11,401.1	1	1	1
Q2	26	9283.2	0.85 (0.51–1.39)	1.04 (0.63–1.71)	1.04 (0.63–1.71)
Q3	74	25,571.2	0.88 (0.60–1.30)	1.12 (0.76–1.67)	1.18 (0.79–1.77)
Q4	19	8792.7	0.65 (0.38–1.13)	0.97 (0.56–1.70)	1.07 (0.61–1.89)
P for trend			0.20	0.80	0.53
CVD					
Q1	201	10,678	1	1	1
Q2	141	8832.2	0.85 (0.68–1.05)	0.99 (0.80–1.24)	1.04 (0.83–1.29)
Q3	318	24,657.4	0.69 (0.57–0.82)	0.83 (0.70–0.99)	0.90 (0.75–1.08)
Q4	91	8536.7	0.56 (0.44–0.72)	0.77 (0.60–0.98)	0.84 (0.65–1.08)
P for trend			< 0.0001	0.009	0.09
CVD mortality					
Q1	43	11,514.4	1	1	1
Q2	22	9395.6	0.63 (0.38–1.06)	0.86 (0.52–1.45)	0.86 (0.51–1.45)
Q3	94	25,817.7	0.99 (0.69–1.42)	1.43 (1–2.06)	1.47 (1–2.15)
Q4	20	8890.7	0.61 (0.36–1.03)	1.08 (0.63–1.84)	1.11 (0.64–1.92)
P for trend			0.38	0.13	0.11
All-cause mortality					
Q1	113	11,514.4	1	1	1
Q2	64	9395.6	0.70 (0.51–0.95)	0.95 (0.70–1.29)	0.89 (0.65–1.22)
Q3	231	25,817.7	0.92 (0.74–1.15)	1.32 (1.05–1.66)	1.19 (0.94–1.51)
Q4	50	8890.7	0.58 (0.41–0.81)	1.01 (0.72–1.41)	0.88 (0.63–1.25)
P for trend			0.035	0.10	0.55

^a^Adjusted for age (year) and sex (men/women).

^b^Additionally adjusted for education (illiterate/primary school/more than primary school), residency (urban/rural), smoking status (never/past/current smoker), daily physical activity (METs-min/day), aspirin use (yes/no), post menopause in women (yes/no), body mass index (kg/m^2^), hypertension (yes/no) and diabetes mellitus (yes/no).

**Table 8:** Multiple adjusted relative risk of global dietary index in cardiovascular diseases among men.

	Mortality rate		Crude model	Model 1^a^ HR (95% CI)	Model 2^b^ HR (95% CI)
	N	Person-year	HR (95% CI)		
MI					
Q1	19	5121.4	1	1	1
Q2	19	4581.2	1.11 (0.59–2.10)	1.29 (0.68–2.45)	1.48 (0.77–2.83)
Q3	44	12,163.6	0.97 (0.57–1.66)	1.17 (0.68–2.02)	1.42 (0.80–2.50)
Q4	16	4786.2	0.89 (0.46–1.74)	1.19 (0.61–2.34)	1.44 (0.72–2.90)
P for trend			0.66	0.66	0.30
CHD					
Q1	32	5121.4	1	1	1
Q2	24	4581.2	0.84 (0.49–1.42)	1.02 (0.60–1.73)	1.12 (0.65–1.92)
Q3	77	12,163.6	1.01 (0.67–1.53)	1.29 (0.85–1.96)	1.44 (0.93–2.23)
Q4	25	4786.2	0.83 (0.49–1.40)	1.22 (0.72–2.08)	1.40 (0.81–2.42)
P for trend			0.77	0.24	0.10
Stroke					
Q1	17	5145.6	1	1	1
Q2	13	4607.1	0.86 (0.42–1.77)	1.12 (0.54–2.30)	1.05 (0.50–2.18)
Q3	37	12,232.6	0.93 (0.52–1.66)	1.29 (0.72–2.31)	1.31 (0.72–2.37)
Q4	10	4799.8	0.64 (0.29–1.40)	1.06 (0.48–2.33)	1.16 (0.52–2.61)
P for trend			0.39	0.60	0.46
CVD					
Q1	113	4727.7	1	1	1
Q2	68	4395.7	0.64 (0.48–0.87)	0.75 (0.56–1.02)	0.80 (0.59–1.09)
Q3	172	11,740	0.61 (0.48–0.78)	0.74 (0.59–0.95)	0.82 (0.64–1.05)
Q4	52	4658.5	0.46 (0.33–0.64)	0.63 (0.45–0.88)	0.70 (0.49–0.99)
P for trend			< 0.001	0.005	0.048
CVD mortality					
Q1	23	5213	1	1	1
Q2	10	4662.9	0.49 (0.23–1.02)	0.68 (0.32–1.42)	0.70 (0.33–1.49)
Q3	58	12,333.2	1.08 (0.66–1.75)	1.62 (0.99–2.63)	1.60 (0.96–2.66)
Q4	14	4853.3	0.66 (0.34–1.28)	1.21 (0.62–2.38)	1.29 (0.64–2.58)
P for trend			0.84	0.08	0.08
All-cause mortality					
Q1	56	5213	1	1	1
Q2	36	4662.9	0.72 (0.47–1.10)	0.98 (0.64–1.49)	0.95 (0.62–1.46)
Q3	144	12,333.2	1.10 (0.80–1.49)	1.60 (1.17–2.19)	1.43 (1.03–1.99)
Q4	25	4853.3	0.48 (0.30–0.77)	0.86 (0.53–1.38)	0.76 (0.46–1.24)
P for trend			0.14	0.14	0.55

^a^Adjusted for age (year).

^b^Additionally adjusted for education (illiterate/primary school/more than primary school), residency (urban/rural), smoking status (never/past/current smoker), daily physical activity (METs-min/day), aspirin use (yes/no), body mass index (kg/m^2^), hypertension (yes/no) and diabetes mellitus (yes/no).

**Table 9:** Multiple adjusted relative risk of global dietary index in cardiovascular diseases among women.

	Mortality rate		Crude model	Model 1^a^ HR (95% CI)	Model 2^b^ HR (95% CI)
	N	Person-year	HR (95% CI)		
MI					
Q1	13	6257.4	1	1	1
Q2	14	4682.2	1.44 (0.68–3.07)	1.76 (0.82–3.75)	1.92 (0.88–4.19)
Q3	25	13,403.1	0.90 (0.46–1.75)	1.11 (0.57–2.19)	1.27 (0.62–2.58)
Q4	6	4021.9	0.72 (0.27–1.89)	1.05 (0.39–2.79)	1.18 (0.44–3.19)
P for trend			0.34	0.92	0.81
CHD					
Q1	16	6257.4	1	1	1
Q2	18	4682.2	1.51 (0.77–2.97)	1.99 (1.01–3.92)	2.11 (1.06–4.22)
Q3	44	13,403.1	1.29 (0.72–2.28)	1.72 (0.97–3.05)	1.90 (1.04–3.47)
Q4	9	4021.9	0.88 (0.39–1.98)	1.45 (0.64–3.31)	1.58 (0.68–3.65)
P for trend			0.95	0.20	0.13
Stroke					
Q1	21	6255.5	1	1	1
Q2	13	4676.1	0.84 (0.42–1.67)	0.97 (0.49–1.95)	1.01 (0.50–2.03)
Q3	37	13,338.7	0.83 (0.49–1.43)	0.99 (0.58–1.71)	1.11 (0.64–1.92)
Q4	9	3992.9	0.68 (0.31–1.48)	0.91 (0.41–2.02)	0.95 (0.41–2.18)
P for trend			0.34	0.89	0.87
CVD					
Q1	88	5950.2	1	1	1
Q2	73	4436.6	1.12 (0.82–1.53)	1.37 (1–1.87)	1.41 (1.03–1.94)
Q3	146	12,917.4	0.76 (0.59–0.99)	0.95 (0.72–1.23)	1.02 (0.78–1.35)
Q4	39	3878.2	0.68 (0.47–0.99)	0.98 (0.67–1.44)	1.06 (0.72–1.57)
P for trend			0.005	0.43	0.87
CVD mortality					
Q1	20	6301.4	1	1	1
Q2	12	4732.7	0.81 (0.40–1.66)	1.14 (0.56–2.35)	1.14 (0.55–2.36)
Q3	36	13,484.4	0.85 (0.49–1.47)	1.21 (0.70–2.09)	1.27 (0.72–2.23)
Q4	6	4037.3	0.47 (0.19–1.18)	0.88 (0.35–2.20)	0.84 (0.33–2.12)
P for trend			0.18	0.81	0.79
All-cause mortality					
Q1	57	6301.4	1	1	1
Q2	28	4732.7	0.66 (0.42–1.04)	0.93 (0.59–1.46)	0.89 (0.56–1.40)
Q3	87	13,484.4	0.72 (0.52–1.01)	1.02 (0.73–1.43)	0.94 (0.66–1.33)
Q4	25	4037.3	0.69 (0.43–1.11)	1.27 (0.79–2.05)	1.18 (0.73–1.91)
P for trend			0.08	0.45	0.77

^a^Adjusted for age (year).

^b^Additionally adjusted for education (illiterate/primary school/more than primary school), residency (urban/rural), smoking status (never/past/current smoker), daily physical activity (METs-min/day), aspirin use (yes/no), post menopause in women (yes/no), body mass index (kg/m^2^), hypertension (yes/no) and diabetes mellitus (yes/no).

## Abbreviations

CVD: Cardiovascular diseases
DQI: Diet Quality Index
DDS: Dietary Diversity Score
HEI: Healthy Eating Index
GDI: Global dietary index
FFQ: Food frequency questionnaire
ICS: Isfahan cohort study
IHHP: Isfahan Healthy Heart Program
MI: Myocardial infarction
CHD: Coronary heart disease
WHO: World Health Organization
IPAQ: International physical activity questionnaire
BMI: Body mass index
SBP: Systolic blood pressure
DBP: Diastolic blood pressure
FBG: Fasting blood glucose
TC: Serum total cholesterol
TG: Triglycerides
HDL-C: High-density lipoprotein cholesterol
ICC: Intraclass correlation coefficient
SCD: Sudden cardiac death
